# Unpacking the impact of COVID-19 on child immunization: evidence from Ghana

**DOI:** 10.1186/s12889-024-19033-4

**Published:** 2024-06-20

**Authors:** Kathrin Durizzo, Koku Awoonor-Williams, Kenneth Harttgen, Isabel Günther

**Affiliations:** 1 ETH Zurich, Clausiusstrasse 37, Zurich, 8092 Switzerland; 2 University of Ghana, P.O. Box LG 13, Legon, Ghana

**Keywords:** COVID-19, Vaccines, Child immunization, Administrative data, Ghana

## Abstract

**Background:**

With the onset of the COVID-19 pandemic, governments implemented social distancing regulations to limit the spread of the disease. Some health experts warned that these measures could negatively affect access to essential health services, such as routine childhood immunizations. Others noted that without these regulations, COVID-19 cases would increase, leading to overburdened health systems.

**Methods:**

We analyze four years (2018–2021) of monthly administrative data on childhood immunizations in all administrative districts in Ghana and exploit variations in social distancing regulations across districts. Given variations in social distancing regulations across Ghanaian districts, we can further differentiate between the effect of public lockdowns and the effect of the pandemic.

**Results:**

We find that child immunizations in Ghana declined by 6% during the public lockdown in April 2020, but the country compensated with higher vaccination rates starting in June, and immunization services recovered to pre-pandemic growth levels by 2021. Time-critical vaccines, such as polio, were not affected at all. We do find a substantially larger disruption in April 2020 (14%) and a slower recovery in 2020 in the 40 lockdown-affected districts. Interestingly, vaccination rates already decreased in February and March by about 5% before the public lockdown and before the pandemic had reached Ghana, but with the pandemic already spreading globally and in the news.

**Conclusion:**

Our results indicate that the negative effect on child immunization was less severe and shorter than predicted by experts. Fear of COVID-19 and delayed vaccination campaigns had a substantial impact on childhood immunization while rising COVID-19 cases and moderate social distancing regulations did not seem to affect immunization rates.

**Supplementary Information:**

The online version contains supplementary material available at 10.1186/s12889-024-19033-4.

## Background

Routine immunizations against childhood illnesses, such as polio, are an essential component of basic health services. Over the last several decades, routine child immunizations have contributed to a decrease in the number of vaccine-preventable illnesses and deaths [1–4]. Despite this progress, ensuring that every child has access to basic immunization services remains challenging in many low- and middle-income countries (LMICs) [5–7] particularly during epidemics and pandemics, such as COVID-19.

Between May and July 2020 the World Health Organization (WHO) conducted a survey with health ministries from more than 100 countries and found that nearly all countries, but particularly LMICs, reported disruptions in basic health services [8]. Routine child immunizations were reportedly among the most frequently disrupted services. The possible reasons for the disruptions include a mix of supply- and demand-side factors. Supply-side factors include overburdened health facilities struggling with interruptions of supply chains of medicines, shifting resources to mitigate the impact of the COVID-19 pandemic, or staff shortages because of sick leave [8]. On the demand side, disruptions might have been linked to fear of COVID-19 infections and the inability to afford health care services because of public lockdowns [7–12]. In particular, health experts argued that social distancing regulations or complete public lockdowns that were swiftly implemented globally by national governments lead to a limitation of outreach and vaccination campaigns on the supply side and difficulties in traveling to health facilities on the demand side [8].

Reported decreases in child immunization rates resulted in major public health concerns in 2020 [13–15], particularly for LMICs [10, 11, 16, 17]. As the WHO report on continuity of essential health services during the pandemic [8] points out LMICs were much more affected by disruptions in essential health services. About 70% of the African, Eastern Mediterranean, and Southeast Asian countries experienced disruptions, whereas only 4% high-income countries reported disruptions [8].. Lack of routine child immunization services can lead to an increase in vaccine-preventable diseases such as diphtheria, measles, or polio [18]. Abbas and colleagues [16] predicted that protecting adults against COVID-19 with social distancing regulations by not maintaining routine child immunizations in Africa would lead to more childhood deaths than COVID-19. In addition, Roberton and colleagues [15] simulated COVID-19-related disruptions of essential children’s health services and access to food in 118 LMICs and found a dramatic excess of 9–45% additional under-five child deaths.

To our knowledge, no study has empirically analyzed the impact of the COVID-19 pandemic on child immunization (i) for an entire LMIC country over 48 months for all essential child vaccines and (ii) differentiating between the impact of social distancing regulations and the pandemic itself. Previous studies only provided evidence on the impact on child immunizations during the lockdown [10, 11, 17, 19], while only one study analyzed the impact of 15 months into the pandemic covering Ethiopia, Laos, Mexico, and South Africa [20]. But this study did not have data on the pre-pandemic yearly trends, hence, the authors could not compare the trend to the two years before COVID-19. Further, to our knowledge, only one study from Sierra Leone analyzed all recommended children’s vaccine types separately, not just the aggregated vaccination coverage rate or a few vaccine types [21]. However, the study from Sierra Leone only provided insights for one single area and only for two months. Most importantly, previous studies could not disentangle the potential reasons for vaccination disruptions because the implementation of social distancing regulations was highly correlated to areas where the numbers of COVID-19 cases were high (e.g., 10, 11, 19). Only, one study used cross-country variance of social distancing regulations to disentangle the potential reasons for vaccination disruptions [20]. The study finds that the stringency of social distancing policies and the COVID-19 incidence is only moderately correlated with health-service disruptions [20],

 To fill the two research gaps mentioned above, which are essential for an optimized policy response to future pandemics, balancing short-term health protection with long-term health impacts, we analyze administrative data from all 260 Ghanaian districts on all 15 recommended child vaccines over four years (January 2018 to December 2021). Using this extensive data allows us to analyze the impact of the pandemic on routine child immunizations for an entire country over 48 months (covering pre-pandemic months and the most severe COVID-19 wave in early 2021), and explore inter-district differences. We compare the effects during the lockdown in April 2020 with later and earlier months to explore the impact of COVID-19 on immunization rates in the short and long term. The fact that Ghana introduced a public lockdown in only 40 out of 260 districts, provides us with a unique case to explore the impact of the lockdown as a mitigation response to the COVID-19 pandemic on immunization services in comparison to the general effects of the pandemic, including rising numbers of COVID-19 cases.

We further created a freely available online dashboard with all descriptive results for policymakers (https://nadel.shinyapps.io/Immunization_Dashboard**).**

## Data and methods

### Context

The first two cases in Ghana were reported on March 12, 2020 ([22]; see Figure A.1 for an overview of the COVID-19 cases in Ghana). To reduce the spread of COVID-19, on March 15, 2020, the Ghanaian government banned all public gatherings such as conferences, festivals, political rallies, and church activities and closed schools ([23]; see Figure A.2 for an overview of the stringency index). On March 29, 2020, the total number of COVID-19 cases in Ghana per day was 152. To avoid an escalation in the number of cases, on March 30, 2020, the government also introduced a geographically concentrated public lockdown in 40 of the most affected districts in the Greater Accra metropolitan area and Greater Kumasi metropolitan area (Figure A.3; [24]). The lockdown banned all non-essential movement, work, and services. Inter-city movement of vehicles and aircraft for private or commercial purposes was forbidden [25]. Traveling to access essential health services, such as routine child immunizations, was still allowed, however, inter-city movement for private or commercial purposes was not allowed [24]. The geographically concentrated public lockdown was lifted on April 19, 2020, while the social distancing and hygienic regulations, such as capacity limitations for events and church services or wearing face masks, remained in effect until March 28, 2022 ([26]; Figure A.2).

After the lockdown was lifted at the end of April 2020, COVID-19 cases in Ghana were still increasing until August 2020 (Figure A.1). Between August and December 2020, the daily number of new confirmed cases dropped considerably. However, starting in December 2020, the number of cases increased again, resulting in a second large wave in January 2021, a third wave in August 2021, and a fourth wave in January 2022 [27]. Vaccination against COVID-19 started on March 1, 2021, for essential staff (such as health workers and frontline executive staff) and populations vulnerable to COVID-19 (such as adults of more than 60 years and people with underlying health conditions).

For our analysis, it is important to mention that a polio vaccination campaign scheduled for April and May 2020 in eight regions in Ghana, as well as a national yellow fever campaign scheduled in April 2020, both had to be postponed to September and October 2020 and to November 2020 [28], respectively, due to the COVID-19 pandemic. The re-launch was only possible since all vaccinators, volunteers, and supervisors were trained on COVID-19 prevention protocols and had been provided with personal protective equipment (PPE) to ensure optimal infection prevention [29].

### Routine child immunization data

We use monthly data from the District Health Information Management System (DHIMS) provided by the Ghana Health Service on 15 vaccine types from January 2018 to December 2021. The monthly values for each vaccine type are available for all 260 districts in Ghana. All indicators measure the number of children (below five years) receiving vaccinations within a given month administered in all health facilities in a given district. We analyze the vaccinations recommended by the Ghana Health Service: Measles-Rubella vaccine (measles-rubella 1, measles-rubella 2), Oral polio vaccine (OPV 0, OPV 1, OPV 2, OPV 3), Pneumococcal vaccine (PCV 1, PCV 2, PCV 3), Pentavalent vaccine (penta 1, penta 2, penta 3), Rotavirus vaccine (rotavirus 1, rotavirus 2), and Yellow Fever vaccine. Although we cannot control for whether the vaccination was given at the recommended time or in the recommended interval (see Appendix S.1), the data provides information on the total number of vaccine doses given in each district over the 48 months between 2018 and 2021. We do not have information on the number of immunizations planned by each district in a given month. However, we assume that the number of vaccinations should grow similar to the yearly population growth rate of children below the age of five. Due to the lack of data at the district level, we assume the same population growth rate for all districts, which was 1.1% annually between 2018 and 2019 [29].

In addition, we use four other datasets to create the following variables: a lockdown variable, a regional COVID-19 cases variable, and district control variables such as monthly number of births, population density in 2020, and poverty rate in 2015. Data on the monthly regional number of COVID-19 cases, COVID-19 cases at the district level in April 2020, and data on public lockdowns in districts in April 2020 are retrieved from Ghana’s outbreak response management updates [30]. The monthly number of births from January 2018 to December 2021 at the district level is extracted from the DHIMS. The estimated population density at the district level for 2020 is from the Ghana COVID-19 monitoring dashboard [31]. The poverty rates at the district level are extracted from the Ghana Poverty Mapping Report 2015 [32]. A more detailed description of the data can be found in Appendixes S1 and S2.

### Statistical analysis

**Fig. 1:**
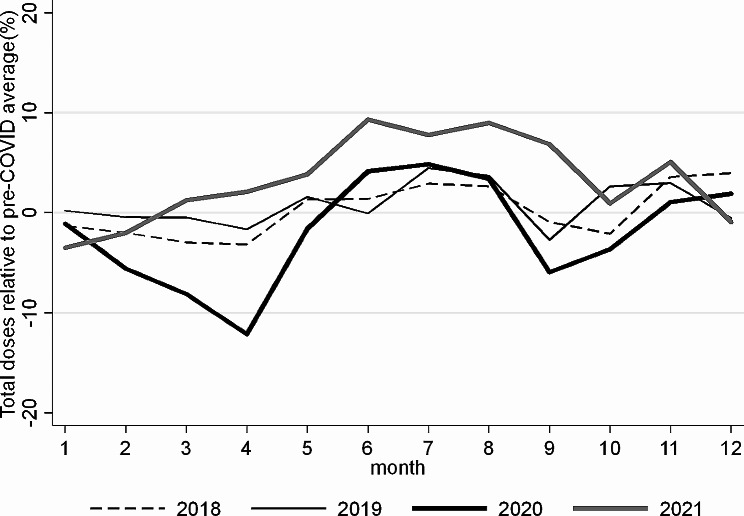
Development of total routine child immunization doses by month Notes: The figure shows the monthly average development of total doses of vaccinations for the years 2018, 2019, 2020, and 2021 relative to the pre-COVID average (2018 and 2019). All results are population growth adjusted. The OLS model analyzing the monthly differences between the years can be found in Table 1

In our analysis, we have a balanced sample with 48 time periods (number of months 2018–2021) and 260 districts in Ghana. Our main outcome variable is the total number of routine child immunizations ($${Y}_{i,m,t})$$ for a district ($$i)$$, month ($$m)$$, and year ($$t)$$, adjusted by a yearly population growth rate of 1.1% (WHO, 2020f). This means that for a nominal change in the number of vaccinations of 1.1%, the calculated real growth rate over time of vaccinations would be 0%. Due to strong seasonal variation in vaccination rates within years (see Fig. 1), we compare only the number of immunizations for the same month over the years or the total yearly changes. Comparing the number of doses from one month to the next within the same year might be highly misleading when evaluating the impact of a pandemic on immunization rates [21, 33, 34].

First, we explore the overall impact of the COVID-19 pandemic on routine child immunization by analyzing the yearly growth rate of the number of total vaccine doses per district from April 2019 to April 2020 as well as from 2019 to 2020 and from 2019 to 2021. The yearly growth rate per district between 2018 and 2019 will serve as a control to understand pre-pandemic patterns. Equation (1) shows how we calculate the growth rate from 2019 to 2020:

1 $${\sum }_{i=1}^{260}\frac{{\sum }_{m=1}^{12}{Y}_{i,m,2020} - \left({\sum }_{m=1}^{12}{Y}_{i,m,2019} * 1.011\right)}{{\sum }_{m=1}^{12}{Y}_{i,m,2019} * 1.011}$$

Next, we analyze the development of routine child immunization by month based on the following OLS regression model with month fixed effects ($${Month}_{i,t})$$, year fixed effects ($$Yea{r}_{i,m}$$), district control variables ($${D}_{i,m,t})$$, and regional COVID-19 cases ($${\beta }_{5}{C}_{i,m,t}).$$ We also include the average number of vaccinations in 2018 and 2019 to control for differences in the levels of vaccinations administered ($${T}_{i})$$. In particular, we estimate the following model:

2 $$\begin{array}{l}{Y_{i,m,t}} = {\beta _0} + {\beta _1}Mont{h_{i,t}} + {\beta _2}Yea{r_{i,m}} + {\beta _3}Mont{h_{i,t}}\\*Yea{r_{i,m}} + {\beta _4}{D_{i,m,t}} + {\beta _5}{C_{i,m,t}} + {\beta _6}{T_i} + {\epsilon _i}\end{array}$$

We introduce the control variables stepwise into the model to test their effect on the coefficients and we use cluster-robust standard errors on a regional level (see Table 1, columns 1 and 2). Moreover, to understand the differences between lockdown- and non-lockdown-affected districts, we run the same model separately (see Table 1, columns 3 and 4). In Table 1 only the coefficient of the interaction term (${Month}_{i,t}\text{*} Yea{r}_{i,m})$ is shown to highlight the monthly effects relative to 2019. The interaction terms with the year 2018 will serve as a robustness test to demonstrate that there were no significant differences before the pandemic (see Table 1, rows 1–11).

**Table 1:** OLS regression of the number of total monthly doses 2018–2021

		Number of doses (1)		Number of doses, with control variables (2)		Number of doses, Non-lockdown-affected districts (3)		Number of doses, Lockdown-affected districts (4)	
Month*Year (ref. 2019)									
	Feburary # 2018	-2.324	[-131.0,126.3]	-31.86	[-171.2,107.5]	45.91	[-52.47,144.3]	-457.9	[-1749.6,833.9]
	March # 2018	-53.15	[-204.0,97.74]	-89.82	[-258.5,78.83]	-82.57	[-254.5,89.33]	-113.3	[-1379.5,1153.0]
	April # 2018	0.730	[-152.0,153.4]	-23.56	[-199.8,152.7]	8.801	[-169.8,187.4]	-201.1	[-1414.2,1012.0]
	May # 2018	67.62	[-76.50,211.7]	57.07	[-105.8,219.9]	-35.13	[-173.5,103.3]	575.7	[-814.1,1965.5]
	June # 2018	160.5	[-59.85,380.9]	158.4	[-87.67,404.5]	62.75	[-110.5,236.0]	700.5	[-595.6,1996.6]
	July # 2018	-1.883	[-268.4,264.6]	-13.74	[-275.2,247.7]	-5.695	[-220.4,209.0]	-54.45	[-1307.9,1199.0]
	August # 2018	25.56	[-228.4,279.5]	41.72	[-198.0,281.4]	-3.314	[-183.5,176.9]	285.4	[-1137.3,1708.0]
	September # 2018	178.1	[-99.15,455.4]	168.1	[-141.6,477.9]	-5.066	[-264.7,254.5]	1135.7	[-329.2,2600.6]
	October # 2018	-173.6	[-442.2,95.10]	-173.5	[-440.0,93.09]	-180.6	[-428.3,67.04]	-123.9	[-1485.1,1237.3]
	November # 2018	115.0	[-212.2,442.2]	131.7	[-185.8,449.1]	23.56	[-215.2,262.4]	744.4	[-965.0,2453.7]
	December # 2018	324.0**	[18.38,629.7]	328.8*	[-28.33,685.9]	115.3	[-187.0,417.7]	1465.9**	[33.14,2898.6]
	Feburary # 2020	-207.2**	[-411.1,-3.388]	-217.0**	[-427.6,-6.479]	-139.3***	[-234.8,-43.74]	-642.1	[-2154.4,870.2]
	March # 2020	-343.1*	[-722.5,36.18]	-364.7*	[-778.3,48.85]	-285.2***	[-485.9,-84.56]	-825.2	[-2434.4,784.1]
	April # 2020	-494.7**	[-864.7,-124.7]	-493.0***	[-831.3,-154.6]	-360.2***	[-515.1,-205.3]	-1361.5*	[-2773.4,50.49]
	May # 2020	-102.0	[-342.8,138.8]	-66.32	[-337.6,204.9]	-168.2**	[-331.4,-5.075]	102.5	[-1442.5,1647.4]
	June # 2020	297.4***	[99.99,494.8]	379.1**	[53.33,704.9]	135.7	[-90.48,362.0]	1129.9	[-442.7,2702.4]
	July # 2020	90.87	[-131.7,313.4]	238.2	[-106.3,582.8]	5.152	[-223.6,234.0]	365.3	[-1198.2,1928.8]
	August # 2020	53.32	[-310.5,417.1]	118.1	[-253.3,489.6]	-20.16	[-430.7,390.4]	345.9	[-1251.9,1943.7]
	September # 2020	-103.9	[-364.5,156.8]	-119.8	[-404.0,164.5]	-231.7	[-529.8,66.39]	366.7	[-1302.6,2036.1]
	October # 2020	-268.9	[-719.8,182.0]	-265.3	[-747.0,216.4]	-227.7	[-537.7,82.37]	-560.9	[-2298.3,1176.6]
	November # 2020	-32.56	[-272.6,207.5]	-11.17	[-290.2,267.9]	-134.3	[-394.2,125.6]	464.5	[-1365.6,2294.6]
	December # 2020	201.2**	[5.755,396.6]	222.1*	[-5.866,450.1]	78.26	[-141.1,297.7]	792.2	[-1020.0,2604.3]
	Feburary # 2021	114.8	[-57.13,286.8]	138.3	[-35.40,311.9]	115.3	[-77.78,308.5]	5.047	[-1563.5,1573.6]
	March # 2021	292.1**	[74.33,509.8]	212.5**	[36.52,388.4]	146.5	[-57.24,350.2]	950.8	[-665.1,2566.7]
	April # 20,221	402.4***	[159.8,645.0]	303.0**	[48.56,557.5]	335.9**	[63.74,608.0]	748.6	[-699.2,2196.3]
	May # 20,221	320.6***	[108.6,532.6]	222.6**	[16.68,428.6]	203.6*	[-38.94,446.1]	981.4	[-676.6,2639.5]
	June # 20,221	705.9***	[395.1,1016.6]	620.6***	[392.3,849.0]	505.6***	[198.1,813.1]	1868.7**	[269.6,3467.8]
	July # 20,221	377.9***	[116.8,639.1]	314.4***	[115.5,513.3]	239.5*	[-0.524,479.6]	1011.7	[-519.2,2542.5]
	August # 2021	484.9***	[189.7,780.1]	501.0***	[187.2,814.9]	338.3**	[19.32,657.3]	1207.6	[-393.3,2808.6]
	September # 2021	715.6***	[281.9,1149.3]	667.0***	[287.8,1046.3]	533.8***	[193.2,874.4]	1706.4**	[74.43,3338.4]
	October # 2021	107.4	[-74.00,288.8]	13.18	[-243.7,270.1]	136.9	[-79.94,353.8]	-81.78	[-1768.1,1604.5]
	November # 2021	313.4**	[26.01,600.8]	210.9	[-50.94,472.7]	229.1*	[-49.45,507.7]	846.1	[-896.5,2588.8]
	December # 2021	177.3	[-305.2,659.7]	162.4	[-382.9,707.8]	22.44	[-315.8,360.6]	915.2	[-665.1,2495.4]
Constant		5400.1***	[4473.2,6327.0]	151.3	[-641.4,944.0]	75.48	[-79.29,230.3]	283.4	[-293.8,860.5]
Month fixed effects		X		X		X		X	
Year fixed effects		X		X		X		X	
Vaccine level				X		X		X	
District control variables				X		X		X	
Regional COVID-19 cases				X		X		X	
Observations		12,480		11,328		9600		1728	
R-squared		0.004		0.922		0.917		0.908	

Note: Only the month and year interaction term of Eq. (2) are shown. Robust regional clustered standard errors were used; 95% confidence interval in parenthesis; * *p* < 0.05, ** *p* < 0.01, *** *p* < 0.001. All results are population growth adjusted. Vaccine level refers to the average number of doses administered in 2018 and 2019 at the district level. District control variables include: the number of births, population density, and poverty rate at the district level. Regional COVID-19 cases refer to the monthly number of COVID-19 cases at the regional level

As a robustness test, we create a five-level lockdown *and* COVID-19 impact status variable ($${I}_{i})$$ indicating if the district was affected by COVID-19 cases at the beginning of the pandemic and how far the district was away from the geographically concentrated public lockdown in April 2020 (for more details, see S2 in the Appendix). For districts very close to the lockdown-affected districts (neighbor districts), we assume that they are very similar in terms of the fear of COVID-19 exposure,^1^ but different in terms of moving restrictions. We estimate the following OLS model for each month separately:

3 $$\begin{array}{l}{Y_{i,m,t}} = {\beta _0} + {\beta _1}Yea{r_{i,m}} + {\beta _2}{I_i} + {\beta _3}{I_i}\\*Yea{r_{i,m}} + {\beta _4}{D_{i,m,t}} + {\beta _5}{T_i} + {\epsilon _i}\end{array}$$

Our coefficient of interest is the interaction term (${I}_{i}\text{*} Yea{r}_{i,m})$ showing how non-lockdown districts relative to lockdown districts developed over the year, controlling for district and year effects (see Table A.1). The months January - March 2020 (see Table A.1, columns 2–4, rows 6–9) will highlight the effects before COVID-19 occurred in Ghana, but when COVID-19 was already spreading globally. The comparison of 2019 with 2018 is again a robustness test to check if there were significant effects before the lockdown (see Table A.2).

Last, we analyze the development of routine child immunization by vaccine type using again Eq. (1), but we run the analysis for each of the 15 vaccine types individually. Additionally, we conduct a series of sensitivity analyses (results available from authors upon request). First, we run Eq. (2) for each of the 15 vaccine types individually to understand if they follow the same trend as the total number of vaccinations. Second, we run Eqs. (2) and (3) with different specifications of ${T}_{i}$, such as the average number of vaccinations from 2018, the average number of vaccinations from 2019, and the lagged number of vaccinations one period before. Lastly, we run all results for non-population growth adjusted numbers.

## Results

### Development of routine child immunization over time and across districts

**Fig. 2:**
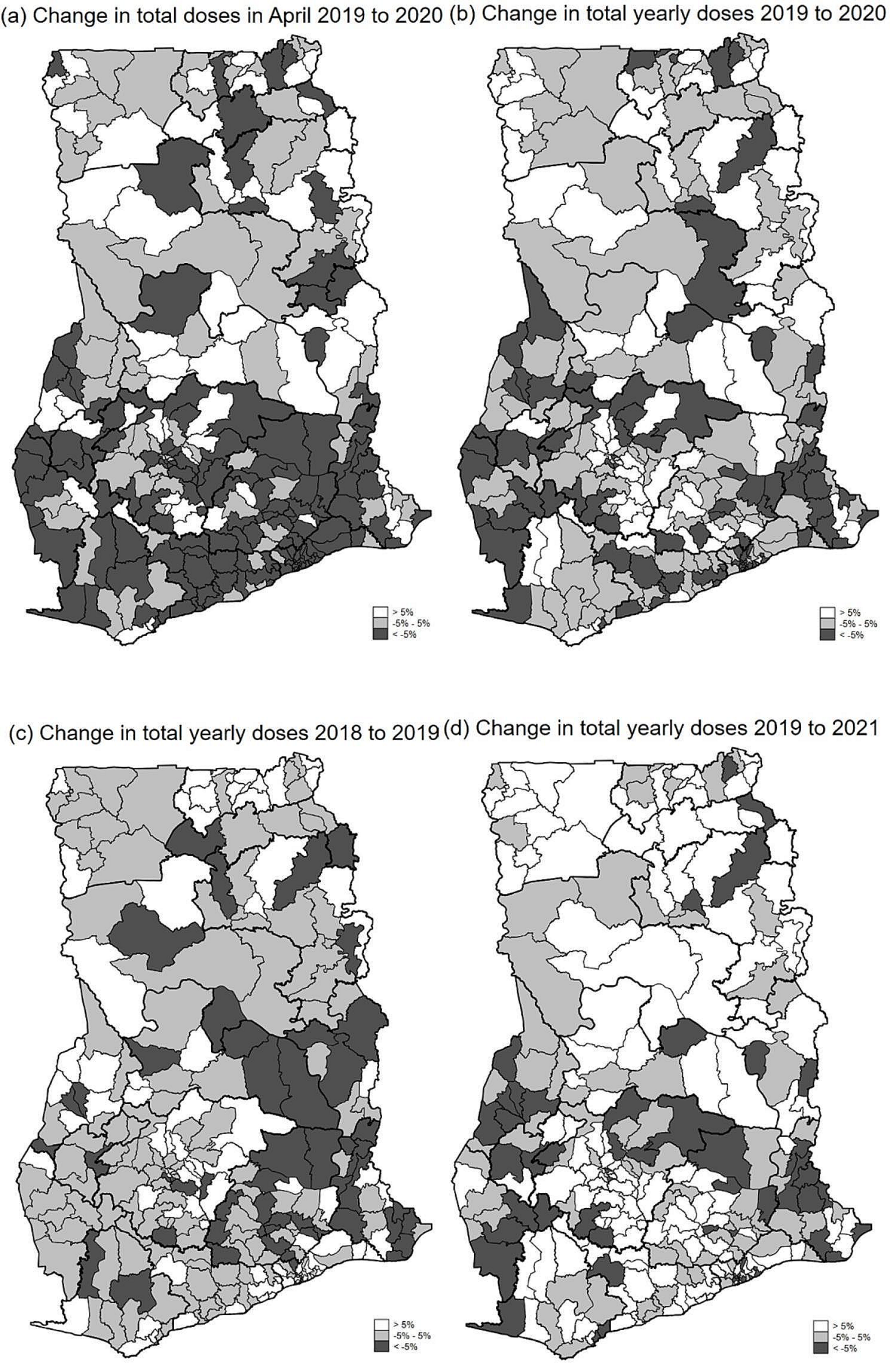
Differences in routine child immunization total doses Notes: Figure (**a**) shows the difference in the total number of administered doses of all children’s routine vaccines per district for April 2020 compared to April 2019. Figure (**b**) shows the yearly total change in the number of doses from 2019 to 2020. Figures (**c**) and (**d**) show the yearly total change in the number of doses from 2018 to 2019 and 2019 to 2021, respectively. All results are population growth adjusted. Detailed results per district and for each immunization type can be found in the online dashboard: https://nadel.shinyapps.io/Immunization_Dashboard/

In April 2020, when a public lockdown was implemented in 40 out of 260 Ghanaian districts (Figure A.3), we found that, on average, child immunizations significantly decreased by -6.4% (95% CI: -8.8 – -4.1) relative to April 2019 across the districts. However, we find a large variance from − 87% to up to 82%: in 138 districts, the number of immunizations dropped by more than 5%, in 67 it stayed about the same (-5% – +5%), and in 55 it increased (Fig. 2a). Comparing the total yearly number of doses administered in 2020 with 2019 (Fig. 2b) indicates that, on average, immunizations decreased only by -1.2% (95% CI: -2.4 – +0.3) across districts. Hence, vaccinations sharply increased after the severe drop in April 2020, even when COVID-19 cases were still rising until July/August 2020. Figure 1 shows that vaccination rates were back to monthly 2019 levels from June 2020 onwards.

Figure 2c further shows that before the COVID-19 pandemic, the yearly number of routine child immunization doses administered increased from 2018 to 2019 by + 1.9% (95% CI: +0.3 – +3.5) per district. However, even before the pandemic, we observed a range in the growth rate of vaccination administered: from − 40% up to more than + 60%. Hence, an analysis of the impact of a shock on immunization rates in a single district might be highly misleading, as even in non-pandemic years immunization rates vary quite substantially from one year to the other within certain districts, whereas on the country level, they may remain constant or slowly increase.

Comparing the pre-pandemic level with the level in 2021 shows that, on average, the total number of doses administered between 2019 and 2021 (Fig. 2d) increased by + 4.2% (95% CI: +2.4 – +6.1) per district over the two years. Figure 1 further shows that the monthly numbers in 2021 again follow the overall seasonal trend of immunization over the year as in 2018 or 2019. This finding indicates that at the national level, the number of doses administered has fully recovered to the pre-pandemic growth levels even during the second and third COVID-19 waves in February 2021 and August 2021 (Figure A.1). Nevertheless, 28 districts (out of 260) still show negative growth in the number of vaccine doses administered of more than − 5% from 2019 to 2021, indicating that not all districts have fully recovered from the COVID-19 shock. These districts should receive a particular policy focus.

All results on district level can be found in the newly created and freely available online dashboard (https://nadel.shinyapps.io/Immunization_Dashboard).

### Impact of fear, COVID-19 cases and social distancing regulations on routine child immunization

Table 1, column 2, analyzes the differences in the number of vaccinations between 2019 and 2020 based on the OLS regression model, controlling for district characteristics and regional COVID-19 cases (see Eq. (2)). While there was a large drop in monthly vaccination rates in April 2020 (-9% or -493 doses, 95% CI: -831.3, -154.6), Table 1, column 2, shows that there was already a decrease in administered doses before COVID-19 first occurred in Ghana in March 2020 and before the public lockdown in April 2020, indicating that the public lockdown only partially explains the severe drop in child immunizations. In particular, we find a 4% drop (-217 doses, 95% CI: -427.6, -6.479) in February 2020 relative to February 2019 and a 7% drop (-364 doses, 95% CI: -778.3, 48.85) in March 2020 relative to March 2019 (see Table 1). Small statistically significant recovery effects can be found already in June (about + 7%., relative to June 2019) at a time with surging COVID-19 cases. In 2021, the results show a significant recovery relative to 2019 for almost all months. The effects also do not substantially change if we no not control for district characteristics as well as regional COVID-19 cases.

We also find a large difference in the effects between the 40 lockdown-affected districts and the districts that did not experience a public lockdown (220 districts): in lockdown-affected districts, the total doses per district from April 2019 to April 2020 decreased on average by -1,361 doses (14%, 95% CI: -2773.4, 50.49) and in not-affected districts by -360 doses (8%, 95% CI: -515.1, -205.3) (see Table 1, columns 3 and 4). The decrease in administered doses in February and March occurred in lockdown- *and* non-lockdown-affected districts.

Table A.1 in the Appendix (columns 2–5) shows that this difference is statistically significant, independent of whether districts had COVID-19 cases or not and independent of whether they were close to districts with a lockdown or not. In January 2020, when China reported the first death from COVID-19, but the pandemic had not yet spread globally, we already found a small significant difference between lockdown-affected districts and their neighbors (Table A.1, column 2). In February 2020, when the first death from COVID-19 outside of China was reported and Italy had a major surge in cases, we already found a larger significant difference between the district types (Table A.1, column 3). Similarly, in March 2020, when the first COVID-19 case occurred in Ghana and the president started with weekly updates on measures taken against the spread of COVID-19, but before the public lockdown was in place, we again found a significant difference between the district types (Table A.1, column 4). Over a year, the statistically significant differences between lockdown- and non-lockdown-affected districts remain (Table A.1, column 1). This indicates that the 141 districts with a negative yearly development from 2019 to 2020 seem to be concentrated in lockdown and COVID-19 impacted districts (49%). Nevertheless, in 2021 relative to 2019, lockdown- and non-lockdown-affected districts are not significantly different from each other over one year.

### Differences in the effects across different vaccine types

**Fig. 3:**
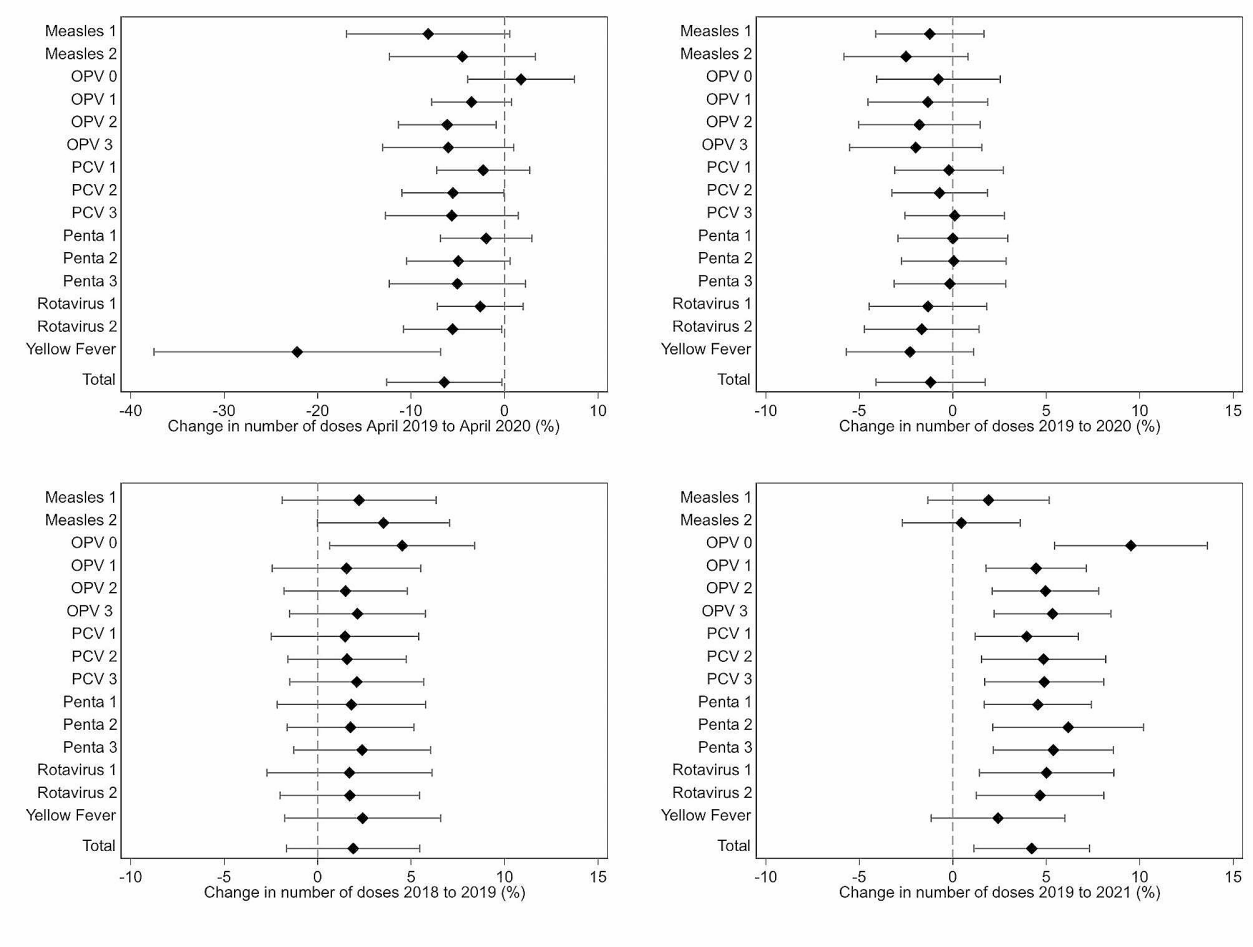
Development of routine child vaccinations by year and type Note: Point estimates (dot) and the 95%-CI (line) are shown. The upper left figure shows the decrease from April 2019 to April 2020, the upper right figure shows the yearly doses decrease from 2019 to 2020, the lower left figure shows the yearly doses from 2018 to 2019, and the lower right figure from 2019 to 2020. All results are population growth adjusted with the 95% confidence interval shown

Similar to the development of total doses, several of the 15 different vaccine types show a substantial drop from April 2019 to April 2020, such as -22.2% (95% CI: -37.5 – -6.8) for yellow fever, -6.1% (95% CI: -11.4 – -0.9) for OPV 2, -5.6% (95% CI: -10.8 – -0.3) for rotavirus 2, and − 5.5% (95% CI: -11.0 – -0.0) for PCV 2 (Fig. 3, upper left panel). For time-critical vaccines, such as OPV 0 and rotavirus 1, we do, however, not find statistically significant changes from April 2019 to April 2020, even though point estimates are slightly negative for most vaccinations. Studying the annual growth rate between 2019 and 2020 for each of the 15 vaccine types reveals a zero growth rate for all vaccinations (Fig. 3, upper right panel). The results highlight that the reasons for vaccination disruptions mentioned in the literature, such as shortages in vaccines and personal protection equipment as well as absenteeism of personnel of the mostly rural health facilities, seem not to be the main driver in Ghana, since OPV 0, which is given at birth in health facilities, seems uninterrupted. On the contrary, delayed vaccination campaigns — as was the case for yellow fever (WHO, 2020f; Gavi, 2020) — seem to play an important role.

## Discussion

We show that during the public lockdown, in April 2020, the total number of child routine immunization doses decreased significantly by -6% (compared to April 2019) across Ghana. This result is substantially lower than what other studies that focused on selected areas of a country observed; for example, reductions of -80% in Karachi, Pakistan [10] and − 84% in one hospital in Sierra Leona [21]. However, we also find large spatial variations in the impact of COVID-19 on vaccinations during the lockdown. If we only focus on the areas with the highest reported COVID-19 cases and the highest population density districts in Ghana, we also find a very large decrease of over − 80% in April 2020 — but this is not consistent across all districts. When we compare the 40 lockdown-affected districts to those with more moderate social distancing regulations (but also with COVID-19 cases), we find that on average the lockdown-affected districts needed longer to recover from the COVID-19 shock and to reach a normal pre-pandemic child vaccination level. Nevertheless, some lockdown-affected areas did not show a decrease in vaccination rates even in April 2020, revealing that even within the same country and with the same restrictions the effect of a lockdown can vary a lot.

Our findings also indicate that the broader way in which COVID-19 impacted child vaccinations is more complex than anticipated. A few months after the lockdown, in June 2020, we found significant compensation and therefore a recovery over the course of 2020. Overall, the yearly number of doses administered in 2020 was similar to 2019 (only 1.2% lower, but not statistically significant). From 2019 to 2021, the number of doses even significantly increased by 4.2%, fully recovering from a pre-pandemic vaccine growth trend. This catch-up effect is substantially faster than expected by health experts [14, 16], especially since COVID-19 cases were still increasing over the same period and the country experienced additional waves of new variants of the disease. Still, 28 out of 260 districts had a negative growth rate between 2019 and 2021.

Additionally, we find that the drop in routine child vaccinations started in February and March 2020, even before the first COVID-19 case occurred in Ghana, and before the implementation of social distancing and public-lockdown measures, but when the disease was already spreading globally. Our findings indicate that uncertainty could have had an important impact on Ghanaian’s protective health behavior. In contrast, we provide evidence that the monthly number of COVID-19 cases in Ghana does not seem to lead to lower routine child vaccination. This finding is in line with the catch-up effect we observed in 2021 — similar to the study from Arsenault and colleagues [20], who show on a country level that COVID-19 incidence is not significantly correlated with health service disruptions. Moreover, we find large variations among the 15 recommended child routine vaccines. We witnessed the largest disruption for yellow fever vaccinations, whereas for time-critical vaccinations that are given at birth (OPV 0), we did not find any significant disruptions — even in lockdown-affected areas in April 2020. This is contrary to studies from other countries that found disruption in time-critical vaccinations given at birth [17, 20, 21]. The findings also suggest that limited access to health facilities was not a driving factor of the disruptions since time-critical vaccinations given at birth were not interrupted. Another reason for the disruptions, mentioned in the literature, is that people cannot afford the health service anymore due to the worsened economic situation [7]. However, we argue that this does not apply to our case, since all routine child vaccinations are given out for free in Ghana. Moreover, we do not find a country-wide shortage of vaccines causing disruptions.

Reasons more likely to drive the disruption in routine child immunizations in Ghana include greater fear of visiting a health facility and delays in vaccination campaigns and outreach sessions of community health workers early in the pandemic. The disruption of vaccinations in February and March 2020 — which started before the first case of COVID-19 was reported in Ghana, but when the pandemic was already widely featured in Ghanaian media — indicates that people were concerned about potential infection when visiting a health facility, and postponed a visit if not absolutely necessary. Disruptions of national vaccination campaigns and of some local outreach programs in the beginning of the pandemic might have also driven short-term disruptions. In particular, the decrease in yellow fever vaccinations in April 2020 could have been due to the national yellow fever campaign that was scheduled in April 2020, but had to be postponed to November 2020 [28, 29, 35]. However, due to the limited data we cannot completely disentangle the effects of these three potential sources. Nevertheless, our results indicate that the decrease in April 2020 was not caused by a generally overburdened health system, but rather a combination of fear and very strict social distancing regulations. Since the number of administered doses largely recovered after two months, even when COVID-19 cases were still rising up until July/August when the 2020 peak was reached, the fear was strongest mainly before or in the beginning of the pandemic and not linked to the actual COVID-19 cases or continuing moderate social distancing regulations.

The study has four main limitations. First, although administrative data from the DHIMS2 platform does not suffer from recall bias [36] nor is it only gathered from a certain subgroup — as is the case for many studies exploring the effects of COVID-19 using survey data [37] or claims data [38] — it does not capture detailed data about the patients. Since we are analyzing the number of vaccine doses administered, we cannot draw any conclusions if the vaccination was given at the recommended time or in the recommended interval. The impact on medical vaccine effectiveness or increased risk of getting infections due to late immunization can therefore not be assessed. Future research will be needed to explore the impact on the quality of care and the impact of potential shifts in vaccination schedules. Second, since we do not have information on the number of immunizations planned by each district in a given month, we assume that the number of vaccinations should grow similarly to the yearly population growth rate of children below the age of five. Compared to other studies not taking population growth into account, our estimates are rather on the conservative side, hence, the faster take-up and recovery may even be under-estimated. Third, despite our statistical approach to disentangle the effects of lockdown- and non-lockdown-affected districts, we cannot rule out that other differences between these districts (apart from COVID-19 cases) might drive our results. Finally, as the large variance of the findings across districts and vaccine types already indicates, the results may not necessarily be the same for other LMICS. More country-wide studies are neededto understand the impact of the pandemic, because studies of single districts or areas, without pre-pandemic vaccination trends, and only for a subset of vaccine types might be misleading.

## Conclusion

This study unpacks the impact of COVID-19 on child immunization and highlights the importance of investigating monthly time series, country-wide, and disaggregated administrative data at the district level. Our results indicate that the negative effect on child immunization was less severe and shorter than predicted by experts. Fear of COVID-19 and delayed vaccination campaigns had a substantial impact on childhood immunization while rising COVID-19 cases and moderate social distancing regulations did not seem to affect immunization rates. The within-country variation we identify in our results emphasizes the need for country-wide data, instead of analyzing only some provinces, in order to fully understand the effect of COVID-19. At the same time, the data should at least be disaggregated at the district level to identify areas that are highly affected by lockdown measures. Our district-level results give policymakers insights into where additional policies or campaigns might be required to target unvaccinated or under-vaccinated children. Our results are therefore important for an optimized policy response to future pandemics, balancing short-term health protection with long-term health impacts.

Future studies that investigate the long-term and country-wide impact of COVID-19 on routine child immunization will show if the positive catch-up effects seen in most districts in Ghana is rather exceptional or if predictions and models have underestimated the reaction time of the healthcare system.

### Electronic supplementary material

Below is the link to the electronic supplementary material.

Supplementary Material 1

## Data Availability

The datasets analysed during the current study are not publicly available due to confidentiality but are available from the corresponding author on reasonable request. The maps and datasets generated on aggregated levels are available https://nadel.shinyapps.io/Immunization_Dashboard.

## Footnotes

1 COVID-19 cases were published only at regional level, so people in each district did not know how much their district was affected [30].
