# Supplementary material for: Unpacking the impact of COVID-19 on child immunization: evidence from Ghana
Source: BMC Public Health. 2024 Jun 20;24:1652. doi: 10.1186/s12889-024-19033-4 (PMC11191367; doi:10.1186/s12889-024-19033-4)
Supplement: Supplementary file 1 — Supplementary Material 1 [file 12889_2024_19033_MOESM1_ESM.docx]

# Supplement

**Figure A.1: Daily new confirmed COVID-19 cases in Ghana since January 1, 2020**

Note: The seven-day moving average was calculated as the average of the daily number and the six lags. The gray shaded area indicates the period of the geographically concentrated public lockdown in Ghana (March 29 to April 19, 2020).

Source: JohnsHopkins (27)—last updated February 8, 2022.

**Figure A.2: Stringency index from Ghana since January 1, 2020**

Note: The Government Response Stringency Index is a composite measure based on nine response indicators, including school closures, workplace closures, and travel bans, rescaled to a value from 0 to 100 (100 = strictest response). This index should not be interpreted as “scoring” the appropriateness or effectiveness of a country’s response; it simply records the number and the strictness of government policies. The gray shaded area indicates the period of the geographically concentrated public lockdown in Ghana (March 29 to April 19, 2020).

Source: Hale and colleagues (36)—last updated February 8, 2022.


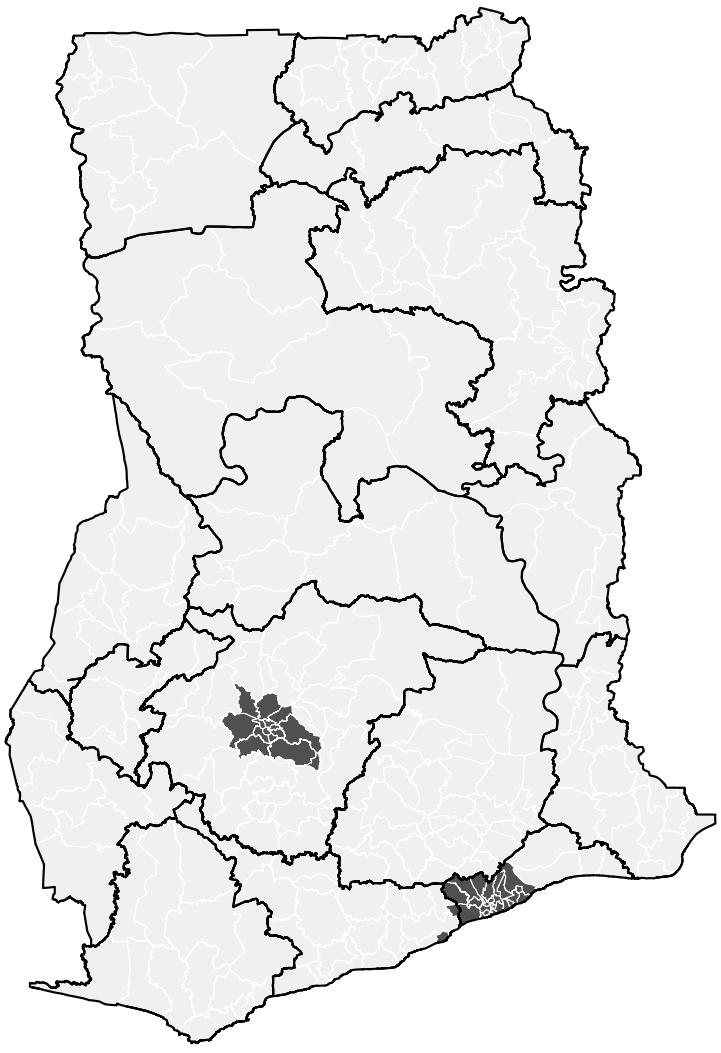


**Figure A.3: Lockdown impact status of districts in Ghana**

Note: Out of the 260 districts in Ghana, 40 districts were under lockdown in April 2020 (dark gray areas) and 220 districts were not affected by the lockdown (light gray areas).

|  | | Number of doses  January – December  (1) | | Number of doses  January  (2) | | Number of doses  February  (3) | | Number of doses  March  (4) | | Number of doses  April  (5) | |
| --- | --- | --- | --- | --- | --- | --- | --- | --- | --- | --- | --- |
| **Year (ref. 2019)** | |  |  |  |  |  |  |  |  |  |  |
| 2020 | | 65.60** | [14.34,116.9] | 160.8** | [0.796,320.8] | -10.80 | [-138.3,116.7] | -111.4 | [-255.9,32.96] | -55.41 | [-191.7,80.84] |
| **Lockdown and COVID-19 impact status**  **(ref. Other districts without COVID)** | |  |  |  |  |  |  |  |  |  |  |
| Lockdown district with COVID | | 828.7*** | [567.4,1090.0] | 611.4* | [-9.432,1232.2] | 753.8* | [-112.1,1619.7] | 759.1* | [-94.98,1613.3] | 954.3** | [179.2,1729.4] |
| Neighbor districts with COVID | | 203.5*** | [116.1,291.0] | -111.5 | [-408.0,184.9] | 194.2** | [14.29,374.1] | 263.4** | [32.32,494.4] | 324.0*** | [111.2,536.9] |
| Neighbor districts without COVID | | 94.70** | [17.98,171.4] | -41.32 | [-283.2,200.5] | 88.96 | [-107.4,285.3] | 88.59 | [-128.0,305.2] | 246.6** | [40.06,453.1] |
| Other districts with COVID | | 171.7*** | [60.76,282.7] | -143.9 | [-351.0,63.08] | -140.8 | [-377.2,95.51] | 107.4 | [-282.8,497.7] | 224.8* | [-37.01,486.7] |
| **Lockdown and COVID-19 impact status *Year** | |  |  |  |  |  |  |  |  |  |  |
|  | Lockdown district with COVID # 2020 | -740.3*** | [-1096.1,-384.5] | -703.9* | [-1542.9,135.1] | -1317.1** | [-2435.2,-198.9] | -1377.9** | [-2547.4,-208.5] | -1983.1*** | [-2951.6,-1014.5] |
|  | Neighbor districts with COVID # 2020 | -114.1 | [-271.5,43.29] | -102.1 | [-505.4,301.3] | -116.0 | [-395.9,164.0] | -161.9 | [-454.4,130.7] | -418.0** | [-740.8,-95.27] |
|  | Neighbor districts without COVID # 2020 | -38.07 | [-142.1,65.95] | -196.1 | [-521.0,128.8] | -53.59 | [-315.1,207.9] | -53.82 | [-340.7,233.1] | -464.6*** | [-807.3,-121.9] |
|  | Other districts with COVID # 2020 | -160.4*** | [-280.6,-40.24] | -17.95 | [-316.6,280.7] | 23.61 | [-294.7,342.0] | -166.2 | [-581.4,249.0] | -421.1** | [-785.7,-56.45] |
| Constant | | 93.68 | [-156.2,343.6] | -111.7 | [-568.6,345.2] | 10.17 | [-581.2,601.5] | 350.7 | [-242.8,944.2] | 130.7 | [-452.0,713.4] |
| Month fixed effects | | X |  |  |  |  |  |  |  |  |  |
| Vaccine level | | X |  | X |  | X |  | X |  | X |  |
| District control variables | | X |  | X |  | X |  | X |  | X |  |
| Observations | | 5664 |  | 472 |  | 472 |  | 472 |  | 472 |  |
| R-squared | | 0.918 |  | 0.957 |  | 0.942 |  | 0.923 |  | 0.946 |  |

**Table A.1: OLS regression of absolute number of total monthly doses 2019-2020 by lockdown and COVID impact status.**

Note: Robust standard errors were used; 95% confidence interval in parenthesis; * p<0.05, ** p<0.01, *** p<0.001. All results are population growth adjusted. Vaccine level refers to the average number of doses administered in 2018 and 2019 at the district level. District control variables include: number of births, population density and poverty rate at district level. Lockdown and COVID-19 impact status fixed effects refer to the five categories “Neighbor of lockdown-affected districts with COVID cases”, “Neighbor of lockdown-affected districts without COVID cases”, “Other districts with COVID cases”, “Other districts without COVID cases” and “Lockdown-affected districts with COVID cases” (see S2 in the Appendix).

|  | | Number of doses  January – December  (1) | | Number of doses  January  (2) | | Number of doses  February  (3) | | Number of doses  March  (4) | | Number of doses  April  (5) | |
| --- | --- | --- | --- | --- | --- | --- | --- | --- | --- | --- | --- |
| **Year (ref. 2019)** | |  |  |  |  |  |  |  |  |  |  |
| 2018 | | 107.2* | [-9.445,223.8] | 206.7 | [-208.3,621.8] | 247.2 | [-143.8,638.1] | 197.4 | [-209.5,604.3] | 194.2 | [-186.5,575.0] |
| **Lockdown and COVID-19 impact status**  **(ref. Other districts without COVID)** | |  |  |  |  |  |  |  |  |  |  |
| Lockdown district with COVID | | 676.7*** | [206.3,1147.1] | 600.8 | [-802.3,2003.9] | 801.0 | [-688.7,2290.7] | 551.5 | [-711.1,1814.1] | 517.8 | [-1019.6,2055.1] |
| Neighbor districts with COVID | | -325.4* | [-662.9,12.07] | -659.0 | [-1765.4,447.5] | -467.3 | [-1616.2,681.5] | -212.7 | [-1288.4,862.9] | -417.7 | [-1486.4,651.1] |
| Neighbor districts without COVID | | -178.9* | [-380.2,22.29] | -257.8 | [-1053.3,537.7] | -198.3 | [-962.3,565.7] | -179.2 | [-959.9,601.4] | -169.2 | [-887.5,549.2] |
| Other districts with COVID | | -345.6* | [-731.0,39.82] | -546.9* | [-1164.9,71.07] | -732.8** | [-1367.8,-97.88] | -531.9 | [-1243.7,180.0] | -613.3* | [-1318.4,91.84] |
| **Lockdown and COVID-19 impact status *Year** | |  |  |  |  |  |  |  |  |  |  |
|  | Lockdown district with COVID # 2018 | -403.1 | [-1220.5,414.2] | -562.7 | [-2646.9,1521.5] | -1056.2 | [-3328.1,1215.7] | -764.3 | [-2740.9,1212.2] | -952.3 | [-3400.5,1495.8] |
|  | Neighbor districts with COVID # 2018 | -272.7 | [-680.5,135.0] | -380.5 | [-1836.0,1075.0] | -324.2 | [-1769.3,1120.9] | -275.4 | [-1624.9,1074.1] | -381.7 | [-1787.5,1024.1] |
|  | Neighbor districts without COVID # 2018 | -172.9 | [-443.5,97.65] | -410.8 | [-1474.1,652.6] | -374.7 | [-1438.8,689.5] | -327.2 | [-1421.7,767.2] | -496.9 | [-1568.6,574.7] |
|  | Other districts with COVID # 2018 | -143.0 | [-372.5,86.48] | -208.7 | [-952.4,534.9] | -61.28 | [-821.2,698.7] | -174.1 | [-979.4,631.2] | -368.8 | [-1142.6,405.0] |
| Constant | | 2475.0*** | [1743.2,3206.8] | 1553.2*** | [722.7,2383.6] | 1778.5*** | [926.4,2630.6] | 1662.8*** | [844.4,2481.2] | 1770.3*** | [783.0,2757.6] |
| Month fixed effects | | X |  |  |  |  |  |  |  |  |  |
| Vaccine level | | X |  | X |  | X |  | X |  | X |  |
| District control variables | | X |  | X |  | X |  | X |  | X |  |
| Observations | | 5664 |  | 472 |  | 472 |  | 472 |  | 472 |  |
| R-squared | | 0.623 |  | 0.732 |  | 0.707 |  | 0.735 |  | 0.672 |  |

**Table A.2: OLS regression of absolute number of total monthly doses 2018-2019 by lockdown and COVID impact status.**

Note: Robust standard errors were used; 95% confidence interval in parenthesis; * p<0.05, ** p<0.01, *** p<0.001. All results are population growth adjusted. Vaccine level refers to the average number of doses administered in 2018 and 2019 at the district level. District control variables include: number of births, population density and poverty rate at district level. Lockdown and COVID-19 impact status fixed effects refer to the five categories “Neighbor of lockdown-affected districts with COVID cases”, “Neighbor of lockdown-affected districts without COVID cases”, “Other districts with COVID cases”, “Other districts without COVID cases” and “Lockdown-affected districts with COVID cases” (see S2 in the Appendix).

## S1: Routine Child Immunization Data

As listed in Table S.1, all children should be vaccinated per Ghana’s recommended immunization schedule. Children with any missed doses should receive “catch-up” vaccines within the first five years of the child’s life. Exceptions are the four time-critical vaccinations: OPV 0, BCG and rotavirus 1 & 2. OPV 0, and BCG should be given at birth or at least within the first two weeks,^[[1]](#footnote-1)^ whereas the rotavirus vaccine at weeks 20 and 24. Most vaccinations require more than one dose for the development of adequate antibody response. A minimum interval of two weeks between each dose is recommended. A longer interval does not necessarily reduce the final antibody response (if the maximum age is adhered to), but it extends the time when the child is at risk of developing the disease. The numbers for the IPV and MenA vaccine will be excluded from our analysis to avoid bias due to the recent introduction of IPV in June 2018 and the anyway decreasing trend of MenA. For BCG, we only received the data until the year 2020 and therefore also excluded it from the analysis. Nevertheless, the results up to 2020 follow the same trend as other time-critical vaccinations, such as OPV 0 (results available from authors upon request).

**Table S.1: Overview of vaccine-preventable diseases targeted by Expanded Programme on Immunization (EPI) in Ghana**

| Disease | Associated Vaccine used in Ghana | Min. Age | Max. Age | Indicator |
| --- | --- | --- | --- | --- |
| Measles | Measles-Rubella vaccine | 9 months | <5years | Measles Rubella 1 |
| Rubella & Congenital Rubella Syndrome |  |  |  | Measles Rubella 2 |
| Poliomyelitis | Oral polio vaccine (OPV) | Birth | <2weeks | OPV/Polio 0 |
|  |  | 6 weeks | <5 years | OPV/Polio 1 |
|  |  |  |  | OPV/Polio 2 |
|  |  |  |  | OPV/Polio 3 |
| Pneumonia, mengitis, other IPD | Pneumococcal vaccine | 6 weeks | <5 years | PCV 1 |
|  |  |  |  | PCV 2 |
|  |  |  |  | PCV 3 |
| Diphtheria | Component of Pentavalent (DPT-Hib-HepB) and Td vaccines | 6 weeks | <5 years | Penta 1 |
| Tetanus | Component of Pentavalent (DPT-Hib-HepB) and Td vaccines |  |  | Penta 2 |
| Pertussis or Whooping cough | Component of Pentavalent (DPT-Hib-HepB) vaccine |  |  | Penta 3 |
| Viral hepatitis | Component of Pentavalent (DPT-Hib-HepB) and HepB vaccines |  |  |  |
| Pneumonia, mengitis, Septicaemia etc. | Component of Pentavalent (DPT-Hib-HepB) vaccine |  |  |  |
| Rotavirus diarrhea | Rotavirus vaccine | 6 weeks | 1^st^ dose: 20 weeks  2^nd^ dose: 24 weeks | Rotavirus 1 |
|  |  |  |  | Rotavirus 2 |
| Yellow fever | Yellow fever vaccine | 9 months | <5 years | Yellow Fever |
| *Tuberculosis (TB)* | *Bacillus Calmette-Guérin (BCG) vaccine* | *Birth* | *<1 year* | *BCG* |
| *Poliomyelitis* | *Inactivated polio vaccine (IPV)* | *14 weeks* | *<5 years* | *IPV* |
| *Meningococcal meningitis* | *Conjugate Meningococcal A (MenAfriVac)Vaccine* | *18 months* | *<5 years* | *MenA* |

Note: Based on the EPI Ghana, for each vaccination the minimum and maximum age for the dose are listed as well as the corresponding indicator in our data.

Source: Gavi (28).

All routine child immunizations are given out for free at all points of care in Ghana, even to those not covered by the National Health Insurance Scheme (NHIS). The distribution of the vaccines is planned based on the Expanded Program on Immunization (EPI) Plan of Ghana and given out (i) in the hospital at all levels of health care, (ii) by outreach of Community-based Health Planning and Services (CHPS), and (iii) by national immunization campaigns in cooperation with the Ministry of Health and health partners. The latter includes only the following vaccine types: measles/rubella, yellow fever, polio and MenA (7, 28, 29).

In line with the general trend of routine child vaccination coverage in Ghana, the data show that almost all vaccinations in 2020 were on a similar level of around 1,100,000 total doses administered within a year in the entire country, corresponding to a coverage rate of around 97% of the target population (29). Although OPV 0 has a lower absolute level (around 900,000 doses yearly), this still corresponds to a high coverage rate of up to 97% since the target population is children younger than two weeks and therefore we expect a smaller number than vaccines for children below five years old. There is a lower absolute level of total doses for measles-rubella 1 & 2, rotavirus 2, and yellow fever, which is again in line with the official WHO-UNICEF coverage rates, corresponding to a coverage rate of around 80% (29). Therefore, we do not assume any bias due to data quality.

## S2: Lockdown and COVID-19 impact status

As stated in Section 2.1, the Ghanaian government introduced a geographically concentrated public lockdown in 40 of the most affected districts in the Greater Accra Metropolitan Area and Greater Kumasi Metropolitan Area from March 30 to April 19, 2020 (Figure A.3; 24).

It is important to note that at that time, not only the lockdown-affected districts had COVID-19 cases, but also 63 additional districts all over the country (Figure S.1, left panel). We do not have access to data on the intensity of COVID-19 cases per district, but we can use the binary information indicating whether a district was affected by COVID-19 in April 2020 that was published in Ghana’s outbreak response management updates (30).

Using a simple OLS regression to analyze which COVID-19 affected districts were put under lockdown reveals that besides the intensity, population density and wealth were also highly correlated. Because the 220 non-lockdown-affected districts significantly differ in terms of population, socioeconomic factors, and urbanization, we also create a three-level categorical variable (Figure S.1, middle panel) to indicate whether the district was affected by the geographically concentrated public lockdown in April 2020, if the district was a first- or second-order neighbor of a lockdown-affected district (with the assumption that these are very similar to lockdown-affected districts in terms of population, socioeconomic factors, and urbanization, as well as fear of COVID-19 exposure, but different in terms of restrictions), and if the district was not affected by the geographically concentrated public lockdown in April 2020 and is further away from a lockdown-affected district. Therefore, we categorize 40 districts as lockdown-affected districts, 45 districts as neighbor districts, and 175 districts as other districts.

Putting together the information on which districts were affected by COVID-19 in April 2020, as well as on which districts were closer to the lockdown hotspot, we created a five-level categorical variable (Figure S.1, right panel) to indicate if a given district was affected by the geographically concentrated public lockdown in April 2020 (40 districts), if a district was a neighbor of a lockdown-affected district and also experienced COVID-19 cases in April 2020 (17 districts), if the district was an “other” district and also experienced COVID-19 cases in April 2020 (46 districts), if the district was a neighbor of a lockdown-affected district but did not experience COVID-19 cases in April 2020 (28 districts), or if the district was an “other” district but did not experience COVID-19 cases in April 2020 (129 districts).

**Figure S.1: Lockdown and COVID-19 affected districts in Ghana**


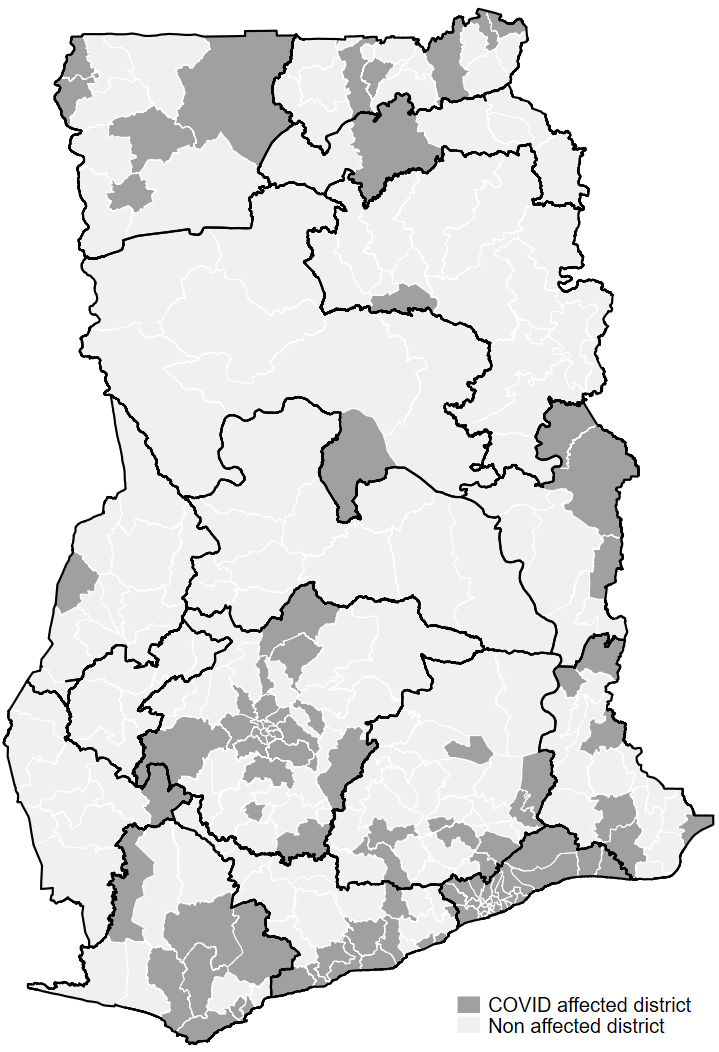

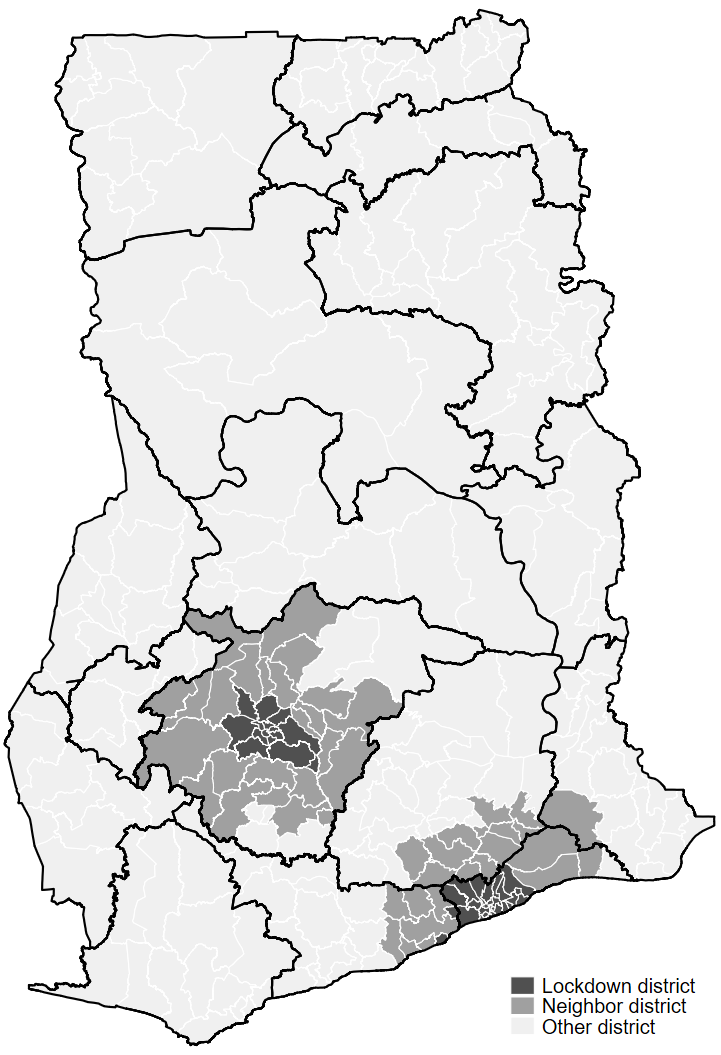

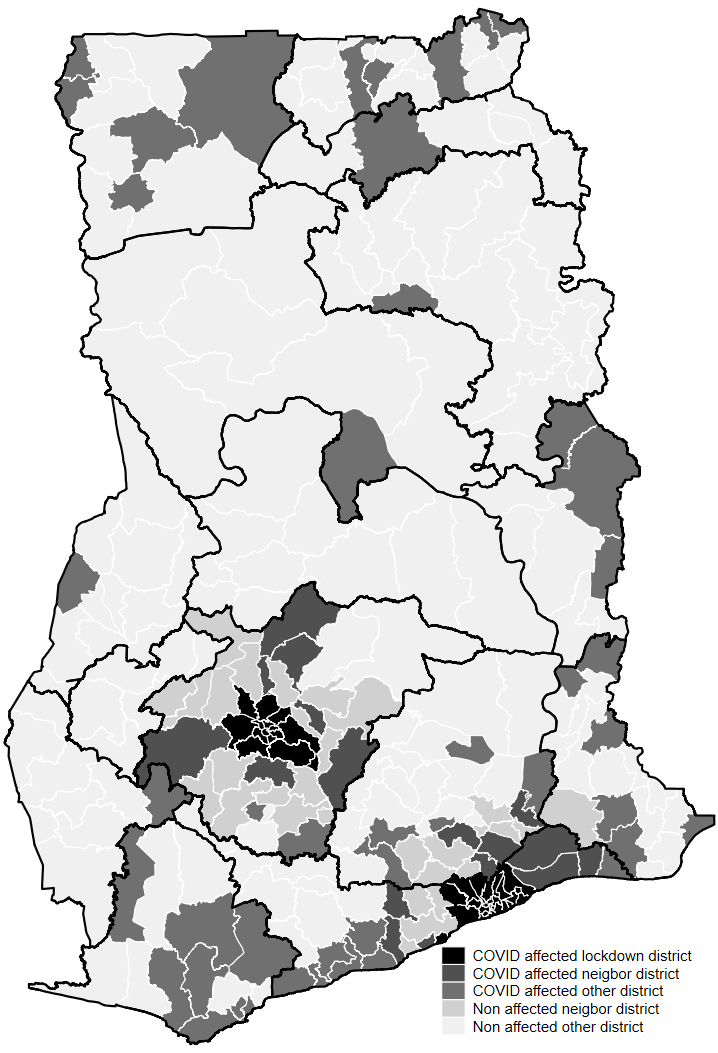


Note: The left figure shows which districts had COVID-19 in April 2020. The middle figure shows in addition to Figure A.3 the neighbor districts (highlighted in light gray). The right figure shows the five-level lockdown and COVID-19 impact status variable.

Source: GHS (30).

1. BCG preferable not beyond two weeks, however, at maximum within the first year. [↑](#footnote-ref-1)
